# The Politics of Attachment: Lines of Flight with Bowlby, Deleuze and Guattari

**DOI:** 10.1177/0263276415605577

**Published:** 2015-10-12

**Authors:** Robbie Duschinsky, Monica Greco, Judith Solomon

**Affiliations:** University of Cambridge; Goldsmiths, University of London; Independent Scholar

**Keywords:** attachment, Deleuze, Guattari, politics, psychology

## Abstract

Research on attachment is widely regarded in sociology and feminist scholarship as politically conservative – oriented by a concern to police families, pathologize mothers and emphasize psychological at the expense of socio-economic factors. These critiques have presented attachment theory as constructing biological imperatives to naturalize contingent, social demands. We propose that a more effective critique of the politically conservative uses of attachment theory is offered by engaging with the ‘attachment system’ at the level of ontology. In developing this argument we draw on Deleuze and Guattari, making use of the common language of ethology which links their ideas to that of attachment theory. The attachment system can and has been reified into an image of the infant returning to their caregiver as an image of familial sufficiency. This has offered ammunition for discourses and institutions which isolate women from health, social and political resources. Yet Deleuze and Guattari can help attachment theory and research to be recognized as a powerful ally for progressive politics, for reflection on the movement of human individuation, and for arguing for the meaningful resourcing of those who care for someone else.

Attachment can be considered ‘perhaps the most important developmental construct ever investigated’ (Sroufe et al., 2005: 51). Not only has it formed the basis for an international research programme, but attachment theory has become an influential perspective on child development and on clinical and welfare practice, and attachment assessments have seen use in these contexts (Kozlowska and Elliott, 2014; Teti and Kim, 2014). Attachment has been described as ‘the most popular theory for explaining parent–child behaviour by professionals and clinicians’ (Barth et al., 2005: 257), and as ‘one of the key concepts in intervention programmes for deprived, neglected and/or maltreated children’ (Van Rosmalen et al., 2014: 24). Beyond social welfare and clinical practice, attachment theory has also influenced other areas of work with children including education (e.g. Cozolino, 2013; Geddes, 2005) and parenting training (e.g. Park, 2012). It has been among the most significant discourses in shaping perceptions of child development and parenting across and beyond Anglophone countries. Yet attachment is widely criticized as the textbook case of a politically conservative research programme, smuggling social norms under the cover of claims to scientific objectivity. Sociologists and feminist scholars have described attachment research as a pretext deployed by clinicians and social care professionals for constructing mothers as solely responsible for infants and then for policing this caregiving. In a study by Hill et al. (1992) of 100 cases of children approved for removal from their families, the attachment behaviour of the child and its perceived implications for their later mental health were cited in every case as part of the rationale. Critiques of attachment theory have also entered public discourse. For example, Hewitt (2013), writing in *The Guardian*, states that ‘parenting manuals based on Bowlby's attachment theory prioritize the bond between mother and child, sideline the father and keep women away from work’.

This article begins by introducing attachment theory as a research paradigm. We then consider the widespread criticism among sociologists and feminist scholars that attachment theory is inherently conservative, and that it constructs biological imperatives to naturalize contingent, social demands. We agree with such critical commentators that there are often serious problems in how attachment theory has sometimes been deployed. We intend to advance these concerns by specifying more precisely the location and significance of any conservative tendency in attachment theory. Our approach is aligned with sociological scholarship which attempts to move beyond social constructionism by considering processes and forces conventionally articulated as ‘biological’ and ‘social’ to be mutually constitutive rather than ontologically distinct (see Davis, 2009, for a review). For instance, scholars have considered topics ranging from patients with medically unexplained symptoms (Greco, 2012) to jewellery designers salivating over gemstones (Hughes, 2011). Yet Gallacher and Kehily (2013) have observed that sociological work outside of a constructionist paradigm on topics relating to developmental psychology has been constrained by the dominance of the ‘new sociology of childhood’. This paradigm rules attention to biological processes as illegitimate, necessarily mechanistic, and politically retrograde. After centuries in which the image of ‘the natural’ has been used to circumscribe the possible structures of society, and particularly the position of women in relation to children, there is legitimate scepticism regarding forms of inquiry which seek to examine the co-production and coupling of biological, social and political assemblages; yet this should not close them off.

In particular, our article responds to calls by sociologists since White (1996) and Rustin (1996) – including Lash (2012) in this journal – for a framework which can encompass the observations of attachment research. Roberts (2013), for example, particularly highlights the importance of research which has found associations between attachment patterns in infancy and early menarche in girls. She alleges that we cede important and highly political ground if attachment theory and research is situated as no more than ideology or merely the product of socio-economic factors, without recognizing an irreducible role for the enmeshment of culture and economics with biology. The urgency of renewed attention to the ontology of attachment has been intensified by recent developments in bringing together attachment and neuroscientific research (e.g. Schore and Schore, 2008). As we have explored elsewhere (Duschinsky et al., 2015), the claim to neuroscientific objectivity has intensified uses of attachment research as a tool for policy discourse on social security and the true nature of citizens.

In reconsidering the ontological stakes of attachment theory, we draw on Gilles Deleuze and Félix Guattari, whose ethological reflections both align with many of the core assumptions of attachment theory, and allow us to present these in a new light. Using Deleuze and Guattari for such work may seem strange to those who associate these theorists with a universal hostility to developmental psychology; yet Deleuze and Guattari (1984 [1971]: 51) specifically affirm the significance of attachment phenomena, and urge that ‘it is not a question of denying the vital importance of parents or the love attachment of children to their mothers and fathers. It is a question of knowing what the place and the function of parents are within desiring-production’. In contrast to most sociological and feminist critiques of attachment theory, Deleuze and Guattari accept attachment as a vital process. However, they demand attention to the ways in which child–parent relationships plug into, affect and are affected by other processes at different levels. In their perspective, biological, social and political assemblages operate below and beyond the level of the human subject. These assemblages constitute the regularities described, and by degrees reified, by the classification system for coding infant–caregiver behaviour in the Ainsworth Strange Situation Procedure, known as the ‘gold standard’ attachment measurement. Beneath these classifications, this article will show the operation, modulation and conflict of lines of flight. Such an account leads to a surprising and far-reaching conclusion: it is attachment phenomena *themselves*, not merely or necessarily attachment theorists, that lend support for conservative political and gender discourses. We argue that the demands for centripetal reunion enjoined by the attachment system frame an image of ‘familial sufficiency disrupted and then regained’. These demands align with and can be deployed as powerful ammunition for discourses and institutions which isolate women from health, social or political resources required for sufficiency.

Genealogical research on attachment theory (Miller and Rose, 1988; Vicedo, 2011) has traced the role of popularizers of Bowlby’s ideas in spreading attachment theory to the ‘psy-disciplines’ – the apparatuses of psychological surveillance and normalization in contemporary society. To this research, we wish to add an account of the potential politics of the assemblage of biological and social components that comprise the attachment system itself – though in recognition that attachment phenomena are realized differently across contexts, and in turn shape these contexts by the way they are accelerated, inhibited or reoriented. Not only, then, would attachment represent a significant developmental construct, but our perspective situates it as a definitive case for showing the need to reassess constructionist approaches to the political formation of human beings. We further contend that coalitions between conservative discourses and the attachment system can best be subject to critique and broken if we scrutinize rather than dismiss the operation of the attachment system, as partly a product of its discursive construction but irreducible to this process. Such scrutiny helps discern the difference between the demands of the attachment system and the health, social and political resources required by a child–caregiver dyad.

## ‘Profoundly Conservative’

Drawing on both his training as a psychoanalyst of the Object Relations school, and contemporary advances in ethological research, the idea of ‘attachment’ and the ‘attachment system’ were introduced by John Bowlby. He described the operation of a disposition in primate infants which directs them to seek proximity to an adult attachment figure when experiencing alarm or separation. Proximity is sought through signals and movements including crying, smiling and crawling. Such behaviour, Bowlby proposed, anticipates a response by this figure which will remove the infant’s experience of potential threat or discomfort. In Bowlby’s account, human infants are born with a capacity, under typical conditions, to develop this disposition to seek the availability of a familiar caregiver when experiencing alarm or separation. However, the emergence of and form taken by this disposition is a composite of neurological, endocrinal, physical and social factors: Bowlby describes the attachment system as a machine dependent for its emergence upon and only effective through the feedback provided by the contingencies of the experience of caregiving. These contingencies in the caregiving environment integrate the different components. Where this particular form of integration does not occur, or occurs only partially, as for instance most starkly in institutionally raised children where no familiar caregiver is available, attachment will not operate as a distinct, integrated behavioural system. Furthermore, reflecting on the contingency of the attachment system and its form, Bowlby (1969: 240–1) hypothesized that the attachment system is inherently incomplete as a system – and this is what makes it work. To operate as a system, attachment behaviour presumes a complementary response in the action of a ‘caregiver behaviour system’ which primes the attachment figure to retrieve the distressed infant. The mesh between attachment and caregiver retrieval systems thus functions to keep an attachment figure near and attentive to the child’s needs: ‘it is fortunate for their survival that babies are so designed by Nature that they beguile and enslave mothers’ (Bowlby, 1958a: 167). He reported that human infants often have their mother as their primary attachment figure, and he emphasized the significance of the mother’s emotional attitude and sensitivity towards her child as integral for development; by contrast ‘little will be said of the father–child relation; his value as the economic and emotional support of the mother will be assumed’ (Bowlby, 1953: 13).

A colleague of Bowlby’s, Mary Ainsworth, formulated a laboratory-based observational measure for assessing individual differences in infant attachment: the Strange Situation Procedure. In formulating this observational measure, Ainsworth was interested in the potential in the life of all infants for anxiety regarding the availability of their familiar caregiver. She had previously studied this potential in the context of other facets of the child’s life in ethnographic research in Ghana and in home observations of Baltimore families. The Strange Situation Procedure was designed to refine and structure the environment so that potential anxiety regarding the availability of the familiar caregiver could come to the fore for observation, in a functional equivalent of instances of separation and reunion embedded in the wider life of the child (see Brown, 2012; Massumi, 1996). It made use of the cues of novelty and separation, which ethological research had suggested to Ainsworth would activate separation distress and attempts to reinstate proximity to the attachment figure. As such, the procedure aimed to mobilize the infant’s visceral expectations based on what happened when anxiety occurred in the past around the availability of the attachment figure, and allow a viewer to interpret these expectations from observed behaviour. As the episodes of the procedure incrementally increase the infant’s anxiety, Ainsworth asked the observer to consider individual differences in the infant’s movement between behavioural systems: the interplay of exploration of novelty and attachment behaviour, in the presence and in the absence of a parent (Ainsworth and Bell, 1970). Though this has been too little discussed in print by attachment researchers, it is important to note that the infant’s response to separation and reunion is not reducible to this set of expectations; for example, it is likely influenced by the caregiver’s facial expression on re-entry, and how frequent infant–caregiver separations are within a culture and what they mean to the adult caregivers (Quinn and Mageo, 2013; Waters and Beauchaine, 2003).

Yet as an index of expectations regarding caregiving experience, the validity of the Strange Situation has stood primarily on the basis of strong associations between individual differences in behaviour in the Strange Situation on the one hand, and observational studies of infants and caregivers at home on the other. Three classifications of infant behaviour were introduced by Ainsworth. Some infants, termed ‘secure’ (B), use their caregiver as a safe base from which to venture off in play. They show distress and seek proximity with their caregiver on reunion, and can be comforted, allowing them to return to play. This behaviour suggests the activation of Bowlby’s attachment system by the anxiety of separation, and its subsidence once the infant feels confident that protection from their attachment figure is available. Ainsworth’s home observations, as well as subsequent research, found that the caregivers for such children were those most sensitive and responsive to the child’s attachment behaviour (e.g. Leerkes, 2011). Other infants, termed ‘insecure-avoidant’ (A), showed little visible affect on separation or reunion with their caregiver – but they were found to have hidden signs of stress, such as a rapid heart-rate (Sroufe and Waters, 1977). Home observations found that the caregivers of these infants tended not to be sensitive and responsive to their attachment behaviour, responding warmly primarily when their child was not distressed (e.g. Isabella and Belsky, 1991). A third pattern was termed ‘insecure-resistant/ambivalent’ (C), and these infants showed distress even before separation and were clingy, frustrated and difficult to comfort on the caregiver’s return, seeming to distrust the availability of the adult even when he or she is present. In contrast to infants classified as ‘secure’ or ‘avoidant’, home observations revealed the mothers of insecure-resistant/ambivalent infants to be relatively unreliable in their response to attachment signals.

Some aspects of Bowlby's and Ainsworth’s work have been extended by later researchers. For example, whereas Bowlby focused on primates, ethologists have documented attachment behaviour in the young and the corresponding caregiving behaviour among mammals and many birds, finding relatively little interspecific variation in these behaviours, even among nest- and den-dwelling species (e.g. Rifkin and Glickman, 2004). Other aspects of Bowlby’s account have been amended by later research, however. For instance, researchers found that infants developed attachments to multiple caregivers early in life, though the more familiar caregiver may still be preferentially discriminated by an infant when alarmed until around the age of two (Umemura et al., 2013). An important amendment to classical attachment theory has been the introduction of a fourth, ‘disorganized/disoriented’ (D), classification by Main and Solomon (1986, 1990). This classification is used when a contradiction or disturbance in the sequencing of the infant’s behaviour suggests that the demands of the attachment system to approach the caregiver are being disrupted by a countervailing, centrifugal affect (e.g. fear, confusion). Infant behaviours coded as disorganized/disoriented include contradictory behaviours or affects occurring simultaneously or sequentially; stereotypic, asymmetric, misdirected or jerky movements; and stilling or freezing. The three Ainsworth infant attachment patterns, as well as the disorganized/disoriented classification, have been found to have associations with measures assessing mental health in later life (Sroufe et al., 2009). For example, Carlson (1998) reported that a classification of disorganized/disoriented attachment in infancy had a strong association with indices of dissociation in adolescence.

Such findings have contributed to the invocation of attachment research in general, and disorganized/disoriented attachment in particular in recent years, within the psy-disciplines and public discourse on childhood. In response, attachment theory and assessments have been situated by sociologists and anthropologists of the family as part of the discursive ‘software’ which operates the ‘hardware’ of the state’s biopolitical surveillance and disciplining of childrearing (e.g. LeVine, 2014). From Oakley (1971) to Koffman (2015), feminist scholars have also described attachment as a theory and research programme animated by a conservative wish to responsibilize women and to police their childrearing, depicted as a matter of the future of the nation. For instance, Contratto (2002: 29, 34) implicates attachment theory as ‘profoundly conservative’, and bent on producing ‘familiar mother-blaming scenarios’. Though a small number of commentators, discussing attachment theory, emphasize its sociological significance but do not allocate a specific political valence (e.g. Redman, 2008), we have not found sociologists or feminists who have argued against its characterization as conservative; this seems to be the consensus. Such a characterization stands in contrast to psychoanalysis, one of the parent disciplines of attachment theory, which has received both criticism and appropriation in the humanities and social sciences. Yet, as Fonagy and Target (2007) have argued, there may be points made by attachment theory which can enrich not only psychoanalysis but also the disciplines which have drawn upon the latter. In particular, they suggest, empirical attachment research may be able to contribute a distinctive and potentially valuable dimension to concerns in a variety of areas concerned with human becoming and relationality (see also Fonagy, 1999; Fonagy et al., 2014).

## Lines of Flight

In setting out to specify further claims regarding the politics of attachment theory, it is important to note that leading contemporary attachment theorists have themselves identified fundamental problems in the way their ideas have been taken up. Much research on attachment, and a great deal of its use in the psy-disciplines, has been premised on the assumption that the four attachment categories – A, B, C or D – form an exhaustive taxonomy, and that placement in the Main and Solomon ‘disorganized/disoriented’ (D) classification necessarily indicates child maltreatment or other forms of dangerously inadequate caregiving. One of the two researchers who introduced the D classification, Mary Main, describes this as a ‘widespread and dangerous’ mischaracterization (Main et al., 2011: 441). The other, one of the authors of this article, urges that disorganization be understood as a dimension which is orthogonal to the A, B and C patterns, reflecting dysregulation in the child–parent relationship. To take attachment behaviour as expressing pre-standing taxonomic categories is to mistake a representation of reality for the reality of the representation. It is a perspective which both depends upon and occludes the dynamic and patterned interplay of biological, social and political forces which *generate* the regularities which the classifications work to pick out. That different patterns of attachment occur in the Strange Situation which are associated with different caregiving experiences shows that, where operational, the attachment system does not operate in a unilateral way; attachment can only operate as a determinate system on the basis of and through the relational ramifications of its mesh with a caregiving system (Solomon and George, 2000, 2011). Attachment theory is a psychology of the interplay of dynamic forces – even though in practice the field has largely spotlighted attachment classifications. Researchers are aware of this discrepancy in a general sense, and some have claimed that the field would benefit from sharper attention to the processes which operate below the level of the classifications, and out of which they are formed. Indeed, a special issue of the journal *Developmental Psychology* was dedicated to affirming that the object of attachment research is not the classifications but the relational phenomena the classifications have been used to describe (see Fraley and Spieker, 2003).

Deleuze and Guattari offer an ontology devised for conceptualizing the generative process which classificatory systems both hide and partially capture. Drawing inspiration from the philosopher and biologist Gilbert Simondon, Deleuze argues that ‘beneath the actual qualities and extensities, species and parts, there are spatio-temporal dynamisms. There are the actualizing, differenciating agencies. They must be surveyed in every domain, even though they are ordinarily hidden by the constituted qualities and extensities’ (Deleuze, 2001 [1968]: 226). To survey and theorize these dynamisms offers a means to further develop the political interrogation of the scene of attachment. It can be pursued by analysing attachment theory’s ‘appeal to biology’ not only in terms of the classifications and prescriptions it makes for human subjects, but also in terms of the coupling of biological, social and political assemblages which operate below and beyond the level of the human subject and which compose it. Deleuze recommends ethological analysis as aligned with this ontology, since it begins with the variety and coupling of semiotic and non-semiotic forces which articulate living forms from within, rather than expressing forms as pre-given essences or structures.

In the *Machinic Unconscious* (2011 [1979])*,* Guattari praises the ethological reflections of Tinbergen, Hinde and Eibl-Eibesfeld for offering an account able to address specific properties and capabilities of semiotic and non-semiotic processes while also being attentive to the variety of configurations that can be enacted. He gives the example, from ethological observations by Immelmann of finches, that:

a diamond female that ‘normally’ does not have a territorial song acquires one as soon as hormones of the male sex are administered to her. She then reproduces the song of the species with which she has been ‘impregnated’ at the time of the ‘sensitive period’ of the first 35 days of her life. (Guattari, 2011 [1979]: 141)

This observational research by Immelmann – which, it might be noted, occurred in a period of collaboration and discussion with Mary Main and later appeared in a jointly edited book (Immelmann et al., 1981) – is used by Guattari to illustrate that just because the bird’s song can be cut loose from the contexts in which we might expect it by circumstances, this ‘by no means implies that it has distanced itself from the most “deterministic” components, like those of apprenticeships through imprint or endocrinal transformations’ (Guattari, 2011 [1979]: 141). While praising such ethological work, however, Guattari cautions against the tendency, which he identifies especially in Tinbergen, to hypostatize the systems that organize sequences of behaviour such that they appear as unitary and unconstituted processes. The danger is that this ‘winds up reconstituting linear causalities’ and contributing to ‘taxonomism’ (2011 [1979]: 115, 146).

Applied to human childhood, Guattari suggests that an ethological perspective recognizes that ‘the child, as an individuated organic totality, only constitutes one intersection among the multiple material, biological, socio-economic and semiotic components which traverse it’ (2011 [1979]: 160). Offering an example which is a good comparison for the coupling of different components in attachment, Guattari gives the case of puberty:

In the life of an adolescent the intrusion of the biological components of puberty is inseparable from the micro-social context within which they appear; they release a series of machinic indexes which have been shown, in addition, to liberate a new abstract machine that will be manifested in the most diverse registers: redirection of perceptive codes, folding of the self and/or poetic, cosmic, social exteriorization, etc. But this release mechanism in reality has nothing unilateral about it because other ‘external’ semiotic components could accelerate, inhibit or reorient the effects of the biological and semiotic components of puberty. (2011 [1979]: 160)

Like the emergence of the biological components of puberty into the subjectivity of the child, the disposition for an infant to seek protection from their caregiver or caregivers when alarmed is not a unilateral mechanism but an assemblage which is realized differently across micro-social contexts, and which in turn shapes varied micro-social contexts in the way it is accelerated, inhibited or reoriented. The individual is, in this perspective, not prior to their environment but codetermined with it, constituted within and through the patterned interaction of affects and movements and changes of bodies in relationships. The implication in terms of how we should understand the ‘attachment system’ is that the reality of such a system, like the classifications through which its variations are described, does not pre-exist the contexts, processes and interactions through which it occurs. As a disposition occurring at the ‘molecular’ level of the neurological system rather than at the ‘molar’ level of the child, the attachment system is thus genuinely underdetermined.

Attachment theory not only can be viewed in ethological perspective but, more than this, it in fact emerged in dialogue with the work of ethologists. For instance, both Tinbergen and Hinde were friends of Bowlby. Considering attachment as an ethological rather than anthropocentric concept, Bowlby (1969: 61) and Main (1979: 641) claim that each child must preserve a line of potential movement to the caregiver from their explorations into the world; whereas other mammals might have burrows or other associated spatial milieus to which they return, primates have determinate figures, living milieus, to whom they always wish to know their line of flight. Deleuze and Guattari agree but immediately consider the possibility that the line of flight does not result in comfort and protection:

A line of flight must be preserved to enable the animal to regain its associated milieu when danger appears. A second kind of line of flight arises when the associated milieu is rocked by blows from the exterior, forcing the animal to abandon it and strike up an association with new portions of exteriority, this time leaning on its interior milieus like fragile crutches. (Deleuze and Guattari, 1987 [1980]: 61)

All children faced with separation and reunion in the Strange Situation are confronted with what Deleuze and Guattari call the possibility of ‘becoming-orphan’, a monadic state cut loose from sustaining ties. It is debatable whether this ultimate possibility is part of the phenomenological experience of an infant who has experienced responsive caregiving; work integrating attachment theory with psychoanalysis has tentatively suggested that infants are predisposed by human evolutionary history to a universal fantasy about the loss of the love-object who is needed for survival (Eagle, 2013; Fonagy, 2001; Slade, 2013). Thinking with Deleuze and Guattari, it can be suggested that the line of flight to the caregiver under situations of alarm indicates a virtual possibility for each infant in the spectre of utter abandonment, which only becomes relative in the actual organization of their particular caregiving environment: ‘absolute deterritorialization becomes relative only after stratification occurs on that plane or body: it is the strata that are always residue, not the opposite’ (1987 [1980]: 63).

## Security, Negation, Dialectic

Considered with Deleuze and Guattari, the Ainsworth ‘organized’ ABC attachment patterns appear as strata, determinate but epiphenomenal, produced by the possibilities for a line of flight which ends in reterritorialization with the caregiver – some manner of return to their safe harbour, rather than exposure to threats reaching potentially all the way to abandonment, injury or death. First we can consider the constellation of forces which comprise what gets observed and classified as ‘secure attachment’ (B), and scrutinize the territorialization and deterritorialization enacted within this pattern. When enacting a line of flight, ‘the child is constructed within a double series’: a set of perceptual relations with the caregiver in the present, and a ‘virtual’ set of experiences, expectations and fantasies from the past (Deleuze, 2001 [1968]: 124). If these series converge such that the infant’s line of flight can overcome or integrate possible obstructions, then the action the infant takes is fully centripetal, directly sending the child to her caregiver when the spectre of absolute deterritorialization threatens. ‘Secure attachment’ appears where this spectre activates a line of flight back to their living milieu. Deleuze and Guattari (1987 [1980]: 61) theorize that where there is reterritorialization there will also be a complementary deterritorialization in a different arena, and vice versa. In line with this conceptualization, confidence in the possibility of safe harbour allows the infant a sense of ‘security’, which holds at bay the spectre of absolute deterritorialization. Such confidence permits the infant to enact a deterritorialization themselves, in the form of excited, expansive and combinatory play.

It can be noted that despite highlighting the radical potential of affirmation and becoming embodied in centrifugal play, Deleuze and Guattari (1987 [1980]: 200) predict for each child that, in the passage into adulthood, both the capacities for exploration and attachment will be mutilated, reconfigured and plugged into the demands of capitalist economics, even if the latter ‘retains some of their debris in well-defined enclosures’. This important critique directly implicates attachment theory and the construct of attachment in so far as they have been used to affirm the affective value of the family at the expense of that of its associated milieu, since for Deleuze and Guattari (1984 [1971]) precisely the family is pivotal to the colonization of desire for capitalist production. The critique of capitalist territorialization (via the family), however, should not be misunderstood as a critique of territorialization per se, or a critique of the ontologically ‘conservative’ vector it represents and of its value in the more general context of desiring-production. The line of flight towards a caregiver is a vector of retreat from the expansive possibilities of experimentation and as such it is itself ‘conservative’. But this conservatism is in turn a vector for the possibility of becoming (as) an individual at the molar level of the child, a vector of the process of returning-to-oneself-as-an-other. As Deleuze and Guattari (1987 [1980]: 178) emphasize:

[y]ou have to keep enough of the organism for it to reform each dawn; and you have to keep small supplies of significance and subjectification, if only to turn them against their own systems when the circumstances demand it.

In this sense, the line of flight represented by a return to safe harbour is much more than a movement of escape from an actual or virtual threat. It is the *line of access* to a reserve of possibilities in excess of those immanent in the situation of play and deterritorialization, through which the child is virtually, and can actually become, more and different than it is (now). The potential vector of movement to an available caregiver, in other words, is what makes possible the eventual ‘escape’ from the caregiver as a source of determination, constraint, stratification. Attachment theorists have addressed this phenomenon with the concept of the attachment figure as a ‘safe base from which to explore’ for the securely attached infant; Ainsworth and Bell (1970: 51), introducing this concept, situated ‘exploration’ and ‘venturing forth’ as ‘equally significant’ to any focus on the solidity of safety and protection. However, the latter has come to dominate in the way attachment theory has been taken up, at the expense of emphasis on the important way that expansive possibilities and play can themselves be fed and innervated by the centripetally oriented attachment system.

In contrast to the situation of the securely attached infant who anticipates a direct line of flight to the caregiver and so can go off to adventure, where a direct return to safe harbour is not possible *negation* and *dialectic* are two logics which can be recruited – as Hegel’s thought illustrates – to serve as alternate strategies for achieving reterritorialization (Deleuze, 1983 [1962]: ch. 5). Such a recruitment yields the constellations of behaviour and affect which comprise the two ‘insecure’ attachment patterns in the Ainsworth Strange Situation. Since Main (1979), attachment theory has often termed these patterns ‘conditional strategies’, a term from evolutionary biology used to refer to the availability of stable alternate routes to a particular outcome, such as reproduction. Where a contradiction occurs between perception (suggesting the caregiver’s physical availability in the room on reunion) and memory (suggesting rebuff or unpredictability from the caregiver when the infant is distressed), then this implies that a direct and stable return to safe harbour is not possible. In such instances the living milieu to which the infant is disposed to return is rocked by rebuff or unpredictability: this requires some leaning on interior milieus, but on the way to a conditional but stable line of flight (Crittenden and Ainsworth, 1989). The result of the play of forces here is a predictable pattern of attachment behaviour, elicited in the Strange Situation.

Where memory suggests that a direct approach will result in rejection by the caregiver and as such be counterproductive in keeping the caregiver available, an insecure-avoidant attachment strategy (A) will shunt the desire for centripetal movement to the caregiver into the interior milieu – as the force of an imperative to negate their wish to regain their living milieu. The infant turns his or her attention to toys or other aspects of the environment as a means of self-distraction, rather than as a site for adventure. Conversely, our perspective suggests that, generating the insecure-resistant/ambivalent (C) attachment strategy, the infant who experiences their living milieu as unpredictable can dialectically utilize the very intensity and unpredictability of their felt distress and frustration to pre-empt, take charge of and give a measure of predictability to the interaction – by throwing a tantrum.

## Conflicting Lines of Flight

All three ‘organized’ Ainsworth patterns respond to the threat of deterritorialization with direct or conditional strategies to achieve the centripetal imperative of the attachment system. No less than attachment theorists, Deleuze and Guattari (1987 [1980]: 297) describe the patterns formed in the interaction between assemblages as occurring upon ‘the plane of organization’, which ‘is constantly working away at the plane of consistency, always trying to plug the lines of flight, stop or interrupt the movements of deterritorialization, weigh them down, restratify them, reconstitute forms and subjects’. Yet as we have seen, Deleuze and Guattari raise the possibility that a second kind of line of flight will arise when the milieu is rocked by blows from the exterior which force the animal to abandon it, ‘leaning on its interior milieus like fragile crutches’. Conflict or problems in the infant’s attachment relationship cannot result in a total abandonment of the attachment figure, since the infant cannot survive on its own. Instead, the result of such difficulties is a disjuncture between a centripetal line of flight to the caregiver, and a countervailing, centrifugal line of flight from the caregiver. Both Deleuze and Guattari, and Main and Solomon in their thinking about attachment disorganization, conceptualize this disjuncture through ethological work on ‘conflict behaviour’. Hinde (1966) had suggested that when animals experience a conflict between incompatible behavioural dispositions, usually the context makes one of these tendencies more salient, and the other waits in abeyance. However, when equilibrium between two behavioural dispositions occurs, Hinde observed what he called ‘conflict behaviour’ – noting contradictory behaviours or affects occurring simultaneously or sequentially; stereotypic, asymmetric, misdirected or jerky movements; and stilling or freezing. Some of these behaviours, he suggested, might be explained by the fact that behaviours and displays of affects could be disinhibited precisely by the contradiction and mutual inhibition of other demands. For instance, when facing a conflict between a tendency to fight an opponent and a tendency to flee in fear, another behaviour system could be activated, such as feeding or drinking.

Discussing ethological observations of ‘conflict behaviour’ (later also called ‘crossroads behaviour’ in Deleuze and Guattari, 1987 [1980]: 368), Guattari (2011 [1979]: 116) notes that ‘during the nuptial parades of birds, abrupt reversals of situation frequently emerge: the courting phase will suddenly be replaced by an aggressive attitude, then simulations of bathing, etc., the various behaviour sequences seeming to be entirely demolished into pieces’. He draws the same conclusion that Main and Solomon will a few years later, and which echoes earlier psychoanalytic thinking about tics and stuttering (e.g. Ferenczi, 1921): that conflict behaviour can be observed in humans experiencing a blockage in action caused by incompatible dispositional imperatives. Of particular importance for such blockages is the role of conflict between those dispositions to act evoked by perceptual relations in the present and those evoked by experiences, expectations and fantasies from the past:

this same mode of semiotization is found in mankind in ‘blockages’, for example, when a person who was accidentally interrupted during the recitation of a text is forced to ‘start over from the beginning’. Behaviour stereotypies are found everywhere in human pragmatic fields, sometimes as ordinary events, other times reflecting chronic impairment as stuttering and phobic or obsessive reiterations will testify. (Guattari, 2011 [1979]: 117)

Guattari proposes that the form taken by conflict behaviour will be determined by a constellation of genetic and environmental factors, and also include ‘improvisation’, and ‘conjunctional tactics’ which attempt to mediate the conflict, as well as express it (2011 [1979]: 134).

Main and Solomon offer the concept of attachment disorganization as an application of Hinde’s ethological reflections to human infant behaviour. The concept was by no means intended to be used, as it has at times by the psy-disciplines, as an ‘abuse category’ of infant behaviour comprised of ‘disorganized/disoriented’ displays. Disorganized/disoriented attachment, as such, must not be regarded as the fourth classification in a neat taxonomy. Such an attempt to resolve reality into representation offers epistemological and moral certainties, but they are quite false. In fact, specifically, Main et al. (1985: 99) state that:

our discovery of the D category of infant Strange Situation behaviour rested on an unwillingness to adopt the ‘essentialist’ or ‘realist’ position regarding the classification of human relationships. It was based on the presumption that both individuals and relationships are unique and that they have a higher ‘reality’ than any classification can fully encompass.

It is notable that Deleuze (1988 [1966]: 69) defends use of the term ‘disorganized’ – not as a synonym for ‘disorder’, but in a precise sense: a disjuncture in the articulation between a human’s perception and their motor-schema of behavioural dispositions formed by the past, such that affects occur which are *incompatible within a behavioural sequence*. This stops the smooth flow of expected behaviour and instead, according to Deleuze and Guattari (1987 [1980]: 179–81), results in confusion, symptoms such as tics or hypochondria, or surprising and potentially ineffective mixes of tendencies towards action. Similarly, the concept of attachment disorganization was defined by Main and Solomon (1990: 133) precisely as ‘an observed contradiction in movement pattern, corresponding to an inferred contradiction in intention or plan’. One such contradiction can occur, as Main and Hesse propose, when the caregiver is themselves a source of alarm: incompatible lines of flight might arise, both towards and away from the caregiver. Yet, contrary to common misconception, they urge recognition that such contradiction need not be caused by abuse (Hesse and Main, 2006). For instance, the presence of multiple social and economic risks experienced by a caregiver can predict high rates of disorganized classifications for their infant, even where there is no known maltreatment (Cyr et al., 2010).

Such findings direct attention to the importance of the social and political context of caregiving: they suggest that the isolation of caregivers from sufficient health, social and political resources can obstruct the capacity of the caregiving system to respond in a sensitive and coherent way with protection and succour to the immature young. Hrdy (2007) has convincingly demonstrated that, though the infant’s attachment system directs them to seek their familiar caregiver when they need support, human caregivers require support from other helpers to maintain a child through a protracted and costly maturation process. She surveys the work of anthropologists, epidemiologists and historians who have documented that, at a population level, increasing the resources available to caregivers from kin and community supports the nurturance they can offer to dependants, and that decreasing such resources directly increases the likelihood that a caregiver will neglect their child. Across these diverse fields of study, researchers have found that the average quality of care to the young fluctuates widely with the resources available to the caregiver. From this we draw the conclusion that if the infant’s constructive grasping of centrifugal possibilities is contingent on the availability of a centripetal line of flight back to a safe harbour, the caregiver’s capacity to offer such a safe harbour, and the quality of care they are able to provide, is similarly contingent on the availability of a line of flight that points away from the child–caregiver dyad. This line of flight connects the caregiver to a reserve of possibilities in excess of those immanent to their caregiving role and, as such, is centripetal relative to *their* individuality, allowing them to return-to-oneself-as-an-other or to *become (as) a caregiver*. To give just one illustrative piece of attachment research which runs counter to stereotypes of the field, a large Australian study found that mothers who valued their career and work identity, regardless of whether or not they returned to work during the first 12 months postpartum, were more likely to have one-year-olds classified as securely attached (Harrison and Ungerer, 2002).

## Concluding Reflections

Beneath the infant attachment classifications, and generating the regularities upon which they are based, occurs the interplay of centripetal and centrifugal forces which open and close the flow of behavioural dispositions. Viewed as such, the properties of the attachment system, working particularly under conditions of alarm (but all the time to some degree) to close down perceived threat through centripetal reterritorialization, mean that this system *itself* can be regarded as supporting its conservative deployment. Indeed, despite his advocacy of its demands, Bowlby fully acknowledged that ‘attachment is fiercely possessive, selfish, utterly intolerant of frustration’ (Robertson and Bowlby, 1950: 138) and that, since women conventionally in our society have responsibility for children, capitulation to the demands of the attachment system for immediate satisfaction would ‘enslave mothers’ (Bowlby, 1958a: 367). The centripetal demands of the attachment system have been championed by conservative policy discourse and also in the psy-disciplines as expressing the needs of the child (see Duschinsky et al., 2015). Yet the distinction between the demands of the attachment system and the needs of the child is one that even infants themselves are making, when they enact conditional strategies (avoidance and ambivalence/resistance) which modulate the demands of the attachment system. Both the allegation that attachment is a fully contingent social construction or a natural process immune from opposition can be countered by attachment as a phenomenon which exemplifies the more general point that:

there is, in the living, an individuation by the individual and not only a functioning that would be the result of an individuation completed once and for all, as if it had been manufactured; the living resolves problems, not only by adapting itself, that is to say by modifying its relation to the environment (which a machine can do), but by modifying itself. (Simondon, 2009 [1989]: 7)

Where the implication of attachment and of attachment research is reduced to the infant’s demand for proximity with their familiar caregiver, then this reification dovetails well with gender and political conservatism. In a society in which women have primary caregiving responsibilities, the attachment behaviour of infants will show a demand for the availability of mothers – and this will appear as support for conservative gender ideologies. Furthermore, the way the attachment system disposes the infant to seek a discriminated, familiar attachment figure as the solution for their distress aligns with interventions which address the behaviour and personality of the parent with primary child care responsibilities – often the mother. This disposition is dramatized by the Strange Situation Procedure, and as such given prominence and visibility. At a macro-level, this aligns with and can be used as rhetorical ammunition for conservative economic ideologies, which treat the emergence of the self-sufficient individual as a process which occurs naturally in families and does not require health, social or political resourcing. The attachment system is well suited to being deterritorialized, reified and incorporated into the rhetorical and affective needs of contemporary capitalism, as we have documented elsewhere using the case of attachment rhetoric within austerity politics in the UK since 2010 (Duschinsky et al., 2015).

Yet when the distinction between the demands of the attachment system and the child’s longer-term needs and potentialities within a caregiver–child dyad are recognized, we do not need to either capitulate to or resent the early imperative of the attachment system. The line of flight towards a caregiver in the face of alarm is indeed a retreat from the expansive possibilities of experimentation, but it can also serve as a vector of the process of returning-to-oneself-as-an-other, as the security offered by knowledge of a safe harbour allows this harbour to be left behind. Such a perspective situates attachment theory and research, not as merely or necessarily conservative, but as a rich repository of observations and reflections for considering the ways that centripetal affects can be resisted and transformed, or used to reassure and bolster centrifugal movement. Studies of the mesh between the attachment and caregiving systems, for example, contradict conservative social discourses. As an example, whereas Bowlby (1958b) argued against day-care on the basis that ‘to deprive a small child of his mother’s companionship is as bad as depriving him of vitamins’, the implications of day-care for young children have been quite thoroughly researched and do not support Bowlby’s position. Indeed, longitudinal research which followed 1153 children from infancy to adolescence has found that quality day-care for young children whose mothers are highly stressed confers a net benefit rather than a risk (NICHD, 1997). Anhert et al. (2004) found that children’s attachments could change from an insecure to a secure classification if the acclimatization process was handled sensitively. Furthermore, only young children who are in day-care for more than 45 hours a week for at least three months have been found to show more behaviour assessed by adults as problematic – and a quarter of them do (see Vandell, 2004, for a review). It appears that unless the extent of the separation is sufficient to undermine the capacity of the child to retain a perception of the availability of the caregiver, attachment research contradicts Bowlby’s claims that maternal care is better or required by ‘Nature’.

Indeed, we disagree with Bowlby in those statements where he claims that society should follow the dictates of ‘Nature’ in order to support the development of children’s psychological health. In general, we do not think that the way biological possibilities articulate with social and political assemblages produces any unitary voice or injunction: the realities are far more complex and our thinking must encompass the full range of forces within which caregiver–child relationships are formed and embedded. We consider that the imperatives of the infant’s attachment system, which prioritize immediate protection as embodied in proximity to the living milieu of the attachment figure, does not always align with the longer-term interests of the child–mother dyad. Like Hrdy, those addressing these issues need to separate out the needs of a caregiver from the imperatives of the attachment system of the young child; whereas the latter demands reunion, the former needs solidarity between adults and sharing of health, social and political resources. The politics of the attachment system might tend to support a conservative agenda, but a perspective which considers the couplings and connections of the attachment system provides resources for a countervailing, progressive politics and form of social analysis.
